# Supplementary material for: Content-rich biological network constructed by mining PubMed abstracts
Source: BMC Bioinformatics. 2004 Oct 8;5:147. doi: 10.1186/1471-2105-5-147 (PMC528731; doi:10.1186/1471-2105-5-147)
Supplement: Additional File 5 — The original Chilibot query results of the term "long-term potentiation (LTP)" and 22 other terms, limiting the latest references analyzed to the years 1990, 1995, 2000, and 2004. [file 1471-2105-5-147-S5.bz2 › chilibotAdditionalFile5/ltp2004/html/KV4.2_ACTIN.html]

 


 **KV4.2** and **ACTIN** 
  
Found 2 abstracts in PubMed,  **2 abstracts were retrieved and analyzed**.  


---

 Search Google  |
 PDF files only 
|  EDU domain only 

---

**Interactive relationship** (e.g. stimulation, inhibition, etc)

- Increased focal  **Kv4.2**  channel expression at the plasma membrane is the result of  **actin**  depolymerization.  Ref: 14551056 Am J Physiol Heart Circ Physiol, 2004
- Our data provide clear evidence that increased numbers and altered distribution of  **Kv4.2**  channels at the cell surface are primarily the result of reorganization of the  **actin**  cytoskeleton.  Ref: 14551056 Am J Physiol Heart Circ Physiol, 2004
- Pretreatment > =1 h of HEK cells with 5 microM cytochalasin D to disrupt the  **actin**  microfilaments greatly augmented whole cell  **Kv4.2**  currents at potentials positive to 20 mV.  Ref: 14551056 Am J Physiol Heart Circ Physiol, 2004
- Here we demonstrate a direct interaction between  **Kv4.2**  and the  **actin**  binding protein, filamin.  Ref: 11102480 J Neurosci, 2000
- Localization and enhanced current density of the  **Kv4.2**  potassium channel by interaction with the  **actin**  binding protein filamin.  Ref: 11102480 J Neurosci, 2000
- Using human embryonic kidney HEK cells as a heterologous expression system, we tested the role of the  **actin**  cytoskeleton in modulating the function of  **Kv4.2**  channels.  Ref: 14551056 Am J Physiol Heart Circ Physiol, 2004

**Parallel relationship** (e.g. studied together, co-existance, homology, etc.)

- We propose that filamin may function as a scaffold protein in the postsynaptic density, mediating a direct link between  **Kv4.2**  and the  **actin**  cytoskeleton,.  Ref: 11102480 J Neurosci, 2000
